# Structural Analysis of G-Quadruplex Formation at the Human *MEST* Promoter

**DOI:** 10.1371/journal.pone.0169433

**Published:** 2017-01-04

**Authors:** Aaron J. Stevens, Martin A. Kennedy

**Affiliations:** Department of Pathology, School of Medicine, University of Otago, Christchurch, New Zealand; Northeastern University, UNITED STATES

## Abstract

The promoter region of the imprinted gene *MEST* contains several motifs capable of forming G-quadruplex (G4) structures, which appear to contribute to consistent allelic dropout during polymerase chain reaction (PCR) analysis of this region. Here, we extend our previous analysis of *MEST* G4 structures by applying fluorescent footprinting techniques to assess non B-DNA structure and topology in dsDNA from the full *MEST* promoter region, under conditions that mimic PCR. We demonstrate that the buffer used for PCR provides an extremely favourable milieu for G4 formation, and that cytosine methylation helps maintain G4 structures during PCR. Additionally, we demonstrate G4 formation at motifs not previously identified through bioinformatic analysis of the *MEST* promoter, and provide nucleotide level resolution for topological reconstruction of these structures. These observations increase our understanding of the mechanisms through which methylation and G4 contribute towards allelic drop-out during PCR.

## Introduction

**Fig 1:**
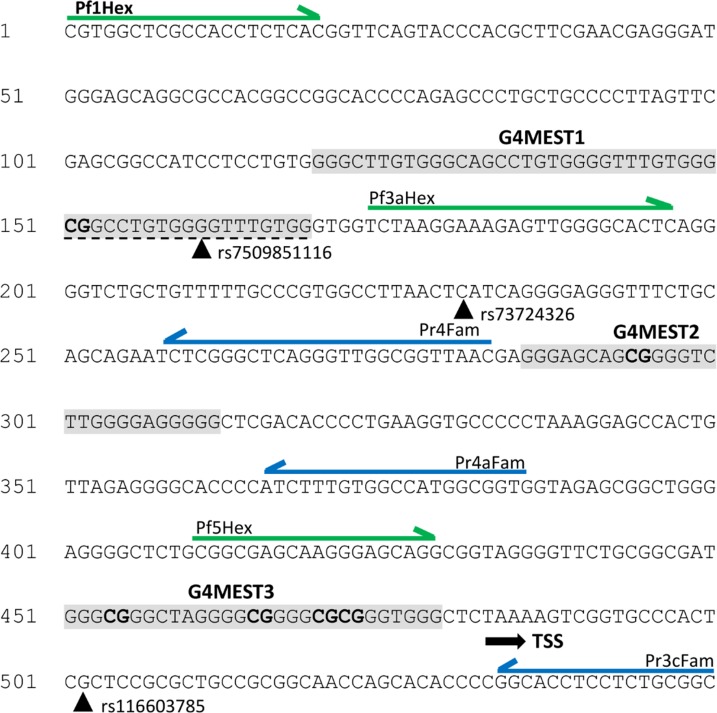
Sequence of MEST promoter region indicating key features. This figure encompasses the hg19 coordinates chr7:130131340–130132187. Features indicated are the three putative G4 sequences (grey shading, with the extended G4MEST1L region indicated by a dashed underline), the three SNPs (arrowheads with rs IDs as indicated), CpG dinucleotides within the G4 regions (bold), the transcription start site (TSS, arrowed), and the fluorescently labelled primers used to generate dsDNA amplicons for FANFA and FADFA (green represents the HEX label and blue represents the FAM label). Nucleotides are numbered at the start of each row.

G-quadruplexes (G4) are DNA structures, prone to forming in guanine rich DNA through Hoogsteen bonding [1]. Although such structures are now recognized to have many biological functions [2–11], they can also present problems for the in vitro analysis of DNA by polymerase chain reaction (PCR) and related techniques [12–14]. Previously, we reported persistent allelic drop-out (ADO) during polymerase chain reaction (PCR) analysis of the human *MEST* promoter region (Fig 1). The *MEST* gene is a maternally imprinted locus that displays paternal expression, with heavy methylation on the maternal allele [15–19], and this allele consistently failed to amplify during PCR. This very robust ADO phenomenon was attributed to the combined effect of non B-DNA structure and DNA methylation in the template DNA [19]. Two key observations provided strong support for this conclusion. First, potassium, which is required for stable formation of G4 in DNA [20, 21], was found to promote ADO of the *MEST* amplicon. Second, 7-deaza dGTP, which prevents formation of the Hoogsteen bonds, alleviated ADO when it was incorporated into synthetic templates prior to PCR [19].

At the *MEST* promoter region, three prominent putative G4 forming regions (G4MEST1L, G4MEST2, and G4MEST3) (Fig 1) were identified bioinformatically and confirmed by analysis of single stranded oligonucleotides using circular dichroism (CD) spectroscopy, and polyacrylamide gel electrophoresis. All three motifs had high thermal stability and displayed characteristics of G4 formation, however, methylation appeared to decrease the G4 stability as measured by CD, which was contrary to our initial hypothesis [19]. It is unclear how cytosine methylation impacts on G4 formation or stability, but this analysis showed that both factors were required to cause ADO and neither G4 formation or DNA methylation in isolation was sufficient.

In this paper we further probe this unusual phenomenon by using fluorescent techniques to map the full non B-DNA forming potential of the *MEST* promoter region in long dsDNA molecules, such as would be present during PCR. We also address several factors that limit our understanding of how methylation and non B-DNA structure contribute towards ADO during PCR of *MEST*. This includes verifying G4 formation in dsDNA, investigating the interaction of methylation on localised G4 formation in dsDNA, and investigating G4 maintenance and re-folding rates during the initial stages of PCR. The overall aim of these experiments was to determine how DNA methylation influences G4 formation and topology at the *MEST* promoter.

## Materials and Methods

This analysis focused on the non B-DNA forming motifs (G4MEST1, G4MEST2 and G4MEST3) that were described by Stevens *et al*. (2014) [19]. Non B-DNA formation was characterised using fluorescence analysis of dimethyl sulfate footprinting assay (FADFA) and fluorescence analysis of nuclease footprinting assay (FANFA), as previously described by Stevens *et al*. (2016) [22].

### Template Synthesis

Non-methylated oligonucleotides (G4MEST1L, G4MEST2, G4MEST3) and methylated oligonucleotides (G4MEST1LM, G4MEST2M, G4MEST3M) were consistent with those previously described by Stevens *et al*. (2014) [19]. For G4MEST3, the situation where each CpG dinucleotide contained 5-methylcytosine was not investigated and requires further analysis. The fluorescent oligonucleotides G4MESTFAM2 and G4MESTFAM3 used in this current study contained FAM labelled markers at the 5’ termini. All oligonucleotides were sourced from Integrated DNA Technologies (IDT Pte. Ltd., Singapore).

Double-stranded DNA templates containing differential fluorescent labels at each terminus were generated using PCR on genomic DNA. In order to cover the full *MEST* promoter region, three primer pairs were used during PCR (Pf1HEX/Pr4Fam, Pf3aHex and Pr4FAM and Pf5HEX/Pr3cFAM) (Table 1, Fig 1). PCR cycling conditions and concentrations were previously described in Stevens *et al*. (2014) [19].

**Table 1:** Oligonucleotides used.

Oligonucleotide name	Oligonucleotide Sequence 5’ to 3’
G4MESTFAM2	/56-FAM/TTAACGAGGGAGCAGCGGGGTCTTGGGGAGGGGG
G4MESTFAM3	/56-FAM/TAGGGGTTCTGCGGCGATGGGCGGGCTAGGGGCGGGGCGCGGGTGGGCTCT
Pf1HEX	/5HEX/CGTGGCTCGCCACCTCTCAC
Pr4Fam	/56-FAM/CGTTAACCGCCAACCCTGAG
Pf3aHex	/5HEX/TCTAAGGAAAGAGTTGGGGCACTCA
Pr4aFAM	/56-FAM/CACCGCCATGGCCACAAAGAT
Pf5HEX	/5HEX/CGGGCGAGCAAGGGAGCA
Pr3cFAM	/56-FAM/TGCCGCAGAGGAGGTGCC
G4MEST1	GGGCTTGTGGGCAGCCTGTGGGGTTTGTGG
G4MEST1L	GGGCTTGTGGGCAGCCTGTGGGGTTTGTGGGCGGCCTGTGGAGTTTGTGGG
G4MEST2	GGGAGCAGCGGGGTCTTGGGGAGGGGG
G4MEST3	GGGCGGGCTAGGGGCGGGGCGCGGGTGGG
G4MEST1A	GAACTTGTGAACAGCCTGTGGAATTTGTGA
G4MEST2A	GAGAGCAGCGAAGTCTTGAAGAGAAAG
G4MEST3A	GAGCGAGCTAGAAGCGAAGCGCGAGTGAG
G4MEST1LM*	GGGCTTGTGGGCAGCCTGTGGGGTTTGTGGGCGGCCTGTGGAGTTTGTGGG
G4MEST2M*	GGGAGCAGCGGGGTCTTGGGGAGGGGG
G4MEST3M*	GGGCGGGCTAGGGGCGGGGCGCGGGTGGG

* Underlining indicates methylated cytosine.

### Cytosine methylation of template DNA

*In vitro* CpG methylation was carried out on double-stranded DNA with M. SssI (New England Biolabs Inc. Ipswich, MA, USA) and was verified using digestion by HpaII and MspII as previously described in Stevens *et al*. (2014) [19]. Phenol:chloroform extraction and ethanol precipitation were then used to remove residual ions and denatured enzyme prior to subjecting the samples to G4 folding conditions.

### Circular dichroism spectroscopy

Unless stated otherwise, CD spectroscopy was performed in 1 x PCR buffer (50 mM KCl, 1.5 mM MgCl_2_), previously described in Stevens *et al*. (2014) [19]. This analysis mimicked a typical PCR cycle, with pre-annealed G4 oligonucleotides subjected to denaturation at 95°C for two minutes, followed by 55°C for fifteen seconds, and 72°C for 45 seconds. Sample temperature was regulated with a Peltier controller and CD spectra were gathered at the end of each temperature step of this mock PCR cycle. The pre-annealing step consisted of denaturation at 95°C for 5 minutes, followed by controlled cooling to room temperature over 4 hours. Oligonucleotides forming G4 structures were then identified and isolated by native polyacrylamide gel electrophoresis, prior to CD spectroscopy.

## Results

### CD Spectroscopy of oligonucleotides

**Fig 2:**
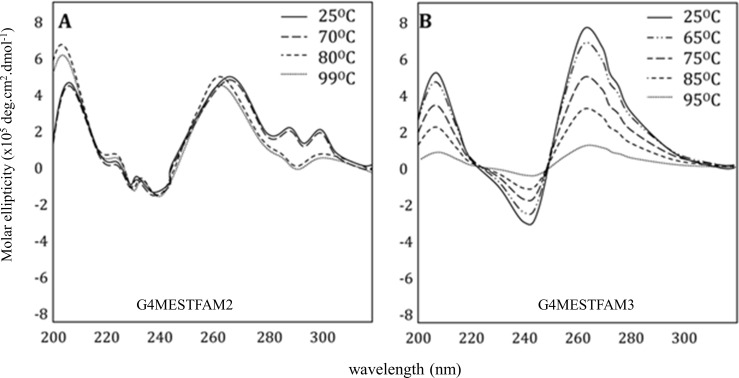
Circular dichroism spectroscopy of FAM labelled single-stranded MEST oligonucleotides. CD spectroscopy was performed in PCR buffer for G4MESTFAM2 (A) and G4MESTFAM3 (B).

As a precursor to DMS treatment the structural conformation and thermal stability of the modified oligonucleotides G4MESTFAM2 and G4MESTFAM3, was investigated using CD spectroscopy in PCR buffer. At 25°C G4MESTFAM2 showed one prominent peak at 260 nm and two smaller peaks at 280 nm and 290 nm, suggestive of mixed parallel and antiparallel G4 conformations (Fig 2A). At 25°C G4MESTFAM3 displayed parallel G4 conformation, represented by a peak at 260 nm and a trough at 240nm (Fig 2B). For both of these oligonucleotides the *T*_m_ and CD spectra were similar to those previously reported [19], confirming that the oligonucleotide modifications did not substantially influence G4 structure.

### FADFA performed on G4MESTFAM2 and G4MESTFAM3 single stranded oligonucleotides

**Fig 3:**
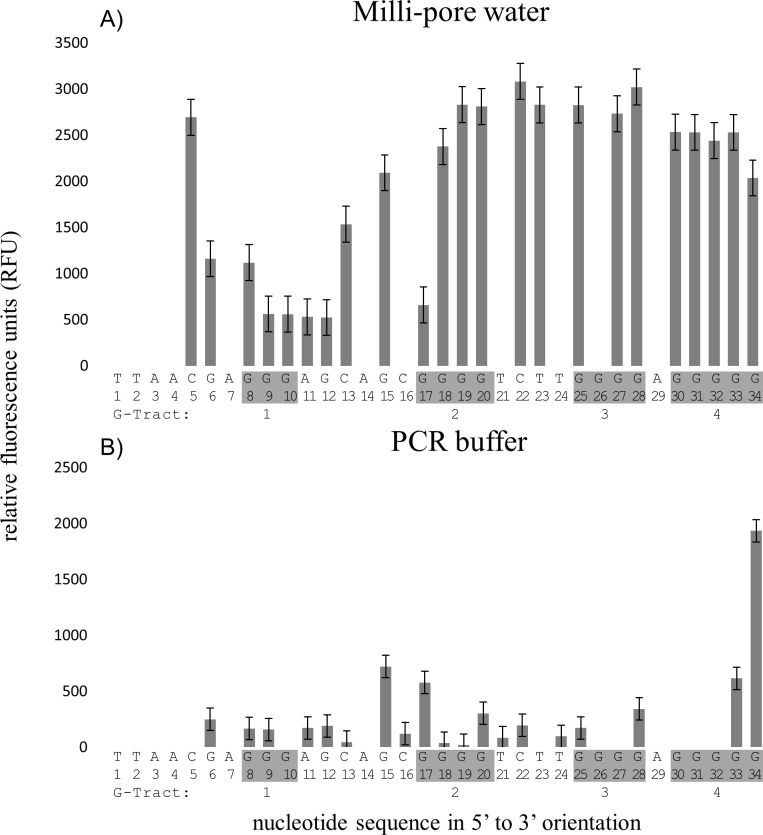
FADFA on single stranded oligonucleotide G4MESTFAM2. Grey bars represent DMS cleavage on FAM labelled template. A. Negative control in Milli-pore water; B. Positive control in PCR buffer containing 1.5 mM MgCl_2_. Grey shading on the sequence indicates G-tracts 1–4 (from left), and nucleotide numbering is indicated below the sequence.

The G4 topology of G4MESTFAM2 and G4MESTFAM3 was investigated using the fluorescence analysis of DMS footprinting assay (FADFA) [22] in PCR buffer (50 mM KCl, 1.5 mM MgCl_2_) (Fig 3). Treatment of G4MESTFAM2 demonstrated reduced cleavage at G-tract 1, the last three guanines of G-tract 2 and the first three guanines of G-tracts 3 and 4. Significant cleavage occurred at the nucleotide positions G15, G17, G28, G33 and G34 (Fig 3). This DMS footprint had a relatively high level of background cleavage and this may reflect the formation of multiple or mixed structures, as indicated by CD spectroscopy (above). It is also likely that G17 and G20 may alternatively contribute towards G4 formation, as indicated by the slightly increased level of cleavage at G20. Excluding MgCl_2_ from the PCR buffer did not appear to have a significant influence on the pattern of cleavage (data not shown).

**Fig 4:**
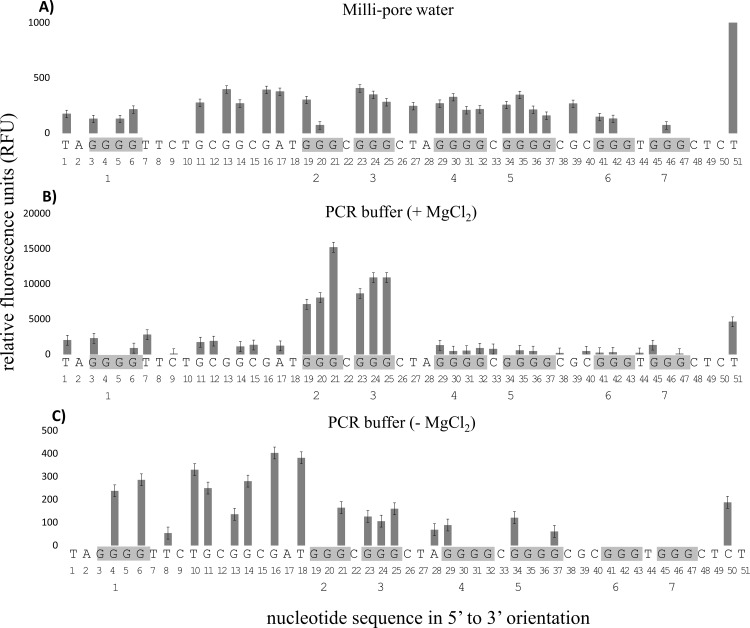
FADFA on single stranded oligonucleotide G4MESTFAM3. Grey bars represent DMS cleavage on FAM labelled template. A: Negative control performed in Milli-pore water; B: DMS footprint for G3MESTFAM3 in PCR buffer with MgCl_2_; C: DMS footprint for G3MESTFAM3 in PCR buffer without MgCl_2_. Grey shading indicates G-tracts 1–4 (from left), and nucleotide numbering is indicated below the sequence.

FADFA performed on the oligonucleotide, G4MESTFAM3 demonstrated complete strand cleavage at G-tracts 2 and 3 and guanine protection at G-tracts 4–7 (Fig 4B). There was insufficient distance between G-tract 1 and the FAM labelled primer for accurate base-calling at these nucleotide positions, however, this G-tract is not predicted by bioinformatic software to contribute towards G4 formation [23]. When MgCl_2_ was excluded from the buffer, the nucleotides G29, G34 and G37 from G-tracts 4 and 5 were additionally cleaved (Fig 4C). Additional patterns of cleavage were also observed for G4MESTFAM3 under alternative buffer conditions that included NaPi and 100 mM NaCl (S1 Fig).

### FANFA performed on dsDNA, after thermal denaturation

DMS footprinting is representative of the averaged pattern of cleavage from many molecules and may not accurately identify G4 regions with high structural polymorphism, such as G4MEST2. We therefore performed an initial screen for G4 formation in dsDNA by the fluorescent analysis of nuclease footprint assay (FANFA), using Mung Bean nuclease [22].

**Fig 5:**
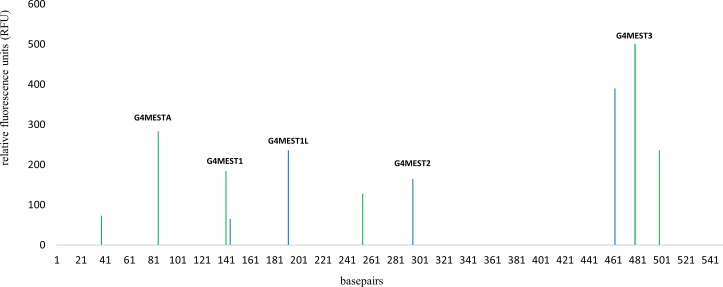
FANFA performed on three templates spanning the full MEST promoter amplicon. Peaks represent regions of single strand digestion on the forward HEX labelled (green) strand and the FAM labelled (blue) reverse strand. Results are the average of three repeat experiments on three separate templates which span the full *MEST* promoter region, compiled into a composite image. Peaks arising from full length, non-digested templates were removed to avoid confusion with cleavage positions. The peak at 255 bp is referred to as G4MESTB (S1 Table). Basepair numbering corresponds with Fig 1.

G4 formation across the full *MEST* promoter region was investigated using three fluorescently labelled primer pairs (Pf1HEX/Pr4Fam, Pf3aHex and Pr4FAM and Pf5HEX/Pr3cFAM) (Table 1) to generate three double stranded DNA amplicons by PCR. These amplicons encompassed the previously characterised motifs, G4MEST1, G4MEST2, and G4MEST3 and were prepared by heat denaturation in PCR buffer. On the G-rich HEX labelled strand, cleavage was observed at a motif which contained an uncharacterised putative G4 region on the complementary strand. This motif is referred to as G4MESTA (85 bp). Additional positions of cleavage corresponded with the 5’ region of G4MEST1 (140 bp), and an additional uncharacterised, putative G4 motif (255 bp) we refer to as G4MESTB. On the C-rich strand (FAM labelled), cleavage corresponded with the 5’ region of G4MEST1L (140 bp), the 3’ region of G4MEST1L (194 bp), G4MEST2 (290 bp) and G4MEST3 (466 bp). The dominant position of cleavage within G4MEST3 corresponded with G-tract 5 (Fig 5).

### FADFA performed on dsDNA after thermal denaturation

Prior CD analysis performed on single stranded DNA demonstrated G4MEST2 and G4MEST3 were stable above 95°C, indicating that G4 are likely to persist through the thermal denaturation stages of PCR [19]. We attempted to use FADFA to test if this observation is consistent in long dsDNA templates. Because we could not directly assess genomic DNA, the assay was performed on the three fluorescent PCR amplicons. The three PCR products individually contained the motifs G4MEST1L, G4MEST2 and G4MEST3, and were subjected to heating at 95°C for two minutes, rapidly cooled to 55°C in a thermal cycler, and immediately treated with DMS.

**Fig 6:**
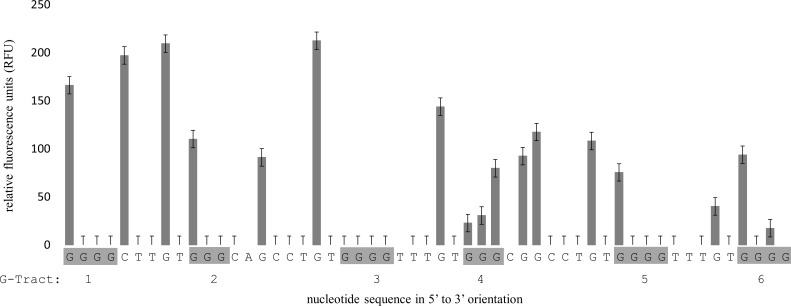
FADFA performed on double stranded G4MEST1L. Grey bars represent DMS cleavage on HEX labelled DNA. The template DNA was heat denatured in PCR buffer (50 mM KCl) prior to DMS treatment. Data shown is the average of three independent replicates. Fluorescent template was generated by PCR using primers PF1HEX and PR4FAM.

**Fig 7:**
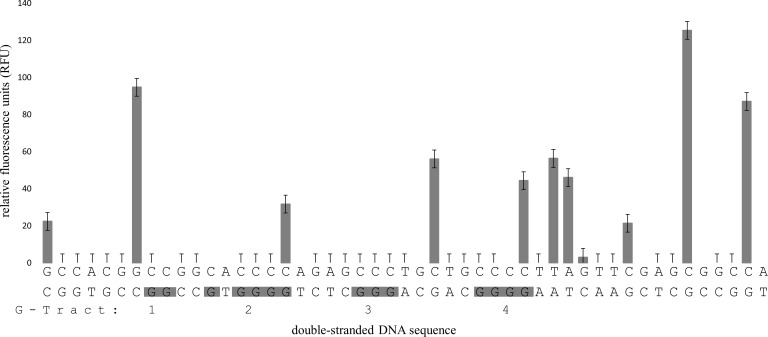
FADFA on G4MESTA. Grey bars represent DMS cleavage on FAM labelled DNA. Grey shading indicates the positions of G-tracts (numbered below), which correspond to the FAM labelled strand. The template DNA was heat denatured in PCR buffer prior to DMS treatment. Data shown is the average of three independent replicates. Fluorescent template was generated by PCR using primers PF1HEX and PR4FAM.

Analysis of the G-rich strand from the 284 bp amplicon that encompassed the region of G4MEST1L demonstrated protection from cleavage at guanine residues from G-tracts 1, 2, 3 and 5 (Fig 6). A low level of cleavage was observed at G-tract 4 suggesting that G-tracts 4 and 5 may be interchangeable in different structures. On the complementary strand (FAM labelled) G4MESTA demonstrated cleavage at the last guanine residues of G-tracts 2 and 4 and at a guanine residing within the link between G-tracts 3 and 4 (Fig 7). The remaining guanine residues within this motif were protected from cleavage, including residue located within the loop of G-tract 1 and 2. This may indicate that this nucleotide contributes towards G4 formation and compensates for the shorter G-tract 1. On the remaining two templates, structural formation was not detected on the C-rich strand (data not shown).

FADFA performed on the 213 bp amplicon that encompassed G4MEST2 had a high level of background cleavage, and therefore did not demonstrate a pattern of guanine protection. This observation is similar to analysis of G4MESTFAM2 in single stranded template (Fig 3) and can indicate either the absence of G4 or the formation of multiple polymorphic non B-DNA structures (data not shown).

**Fig 8:**
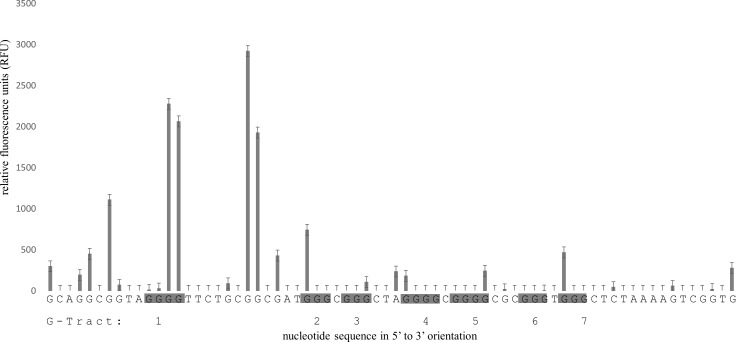
FADFA on G4MEST3. Grey bars represent DMS cleavage at guanine residues on FAM labelled DNA. The template DNA was heat denatured in PCR buffer prior to DMS treatment. Data shown is the average of three independent replicates. Fluorescent template was generated by PCR using primers PF5HEX and PR3cFAM.

FADFA analysis of G4MEST3 demonstrated guanine protection at G-tracts 2 through to 7 (Fig 8). This pattern of DMS protection was unique because it spanned six G-tracts, however, it was highly reproducible.

### FADFA performed on dsDNA, without thermal denaturation

We next investigated if G4 structure in dsDNA could out-compete existing Watson-Crick base pairs without thermodynamic strand denaturation. The aim of this analysis was to test if thermal denaturation during PCR promoted G4 formation by separating dsDNA, or if G4 motifs were already pre-formed in genomic DNA. The absence of G4 formation at the outset of this experiment was validated using FADFA (data not shown), after which, the templates were suspended in PCR buffer and incubated at 37°C for 24 hours. After incubation, the templates were re-assessed for G4 formation using FADFA.

**Fig 9:**
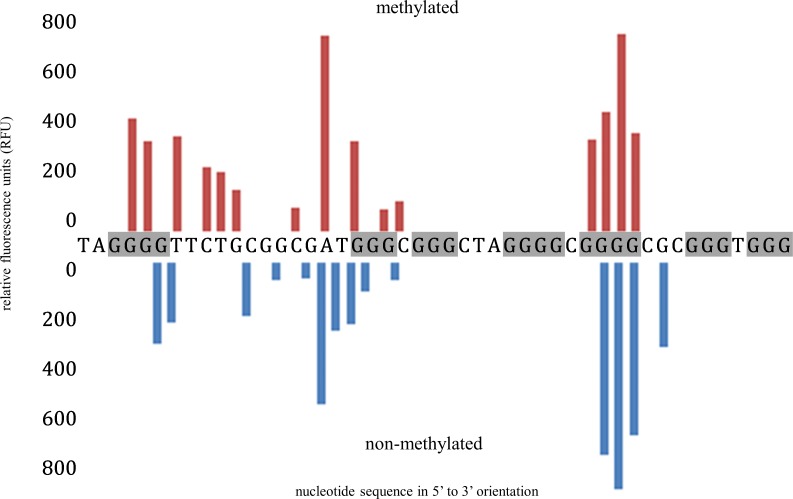
FADFA on double stranded template containing G4MEST3, without prior denaturation. DNA sequence is presented in 5’ to 3’ orientation, showing the pattern of guanine cleavage throughout the region of G4MEST3 on the forward (G-rich), HEX labelled strand. Cleavage of methylated DNA is represented by red bars (upper graph) and of non-methylated DNA by blue bars (lower graph). Fluorescent template was generated by PCR using primers PF5HEX and PR3cFAM.

The only motif to demonstrate a pattern of guanine protection representative of G4 formation after this treatment was G4MEST3 (Fig 9). This indicated that the motif of G4MEST3 had transitioned from a canonical B-DNA state to adopt a G4 structure, without thermal strand denaturation. G-tracts 3, 4, 6 and 7 were protected from guanine cleavage, whereas G-tracts 1, 2 and 5 were cleaved. This experiment was then repeated on methylated template, to test whether cytosine methylation influenced G4 folding. The pattern of cleavage at G4MEST3 was consistent for both methylated and non-methylated templates, demonstrating that cytosine methylation did not significantly influence either the ability to adopt G4 structure, or the G4 conformation under these conditions (Fig 9).

### G4 structure formation during a typical PCR cycle

To further investigate how the conditions of PCR may promote G4 formation and cause ADO of methylated DNA, we collected CD spectra from methylated and non-methylated *MEST* G4 at each of the three key steps of a mock PCR cycle. In this analysis, the spectral profiles of the (non-fluorescent) oligonucleotides G4MEST1L, GMEST2 and G4MEST3 [19] were compared to their methylated counterparts (G4MEST1LM, G4MEST2M and G4MEST3M) in PCR buffer, containing 1.5 mM MgCl_2_ and 50 mM KCl. Spectral profiles were first gathered at 25°C, then after two minutes at 95°C (denaturation), after 15 secs at 55°C (annealing), and after 45 secs at 72°C (extension).

**Fig 10:**
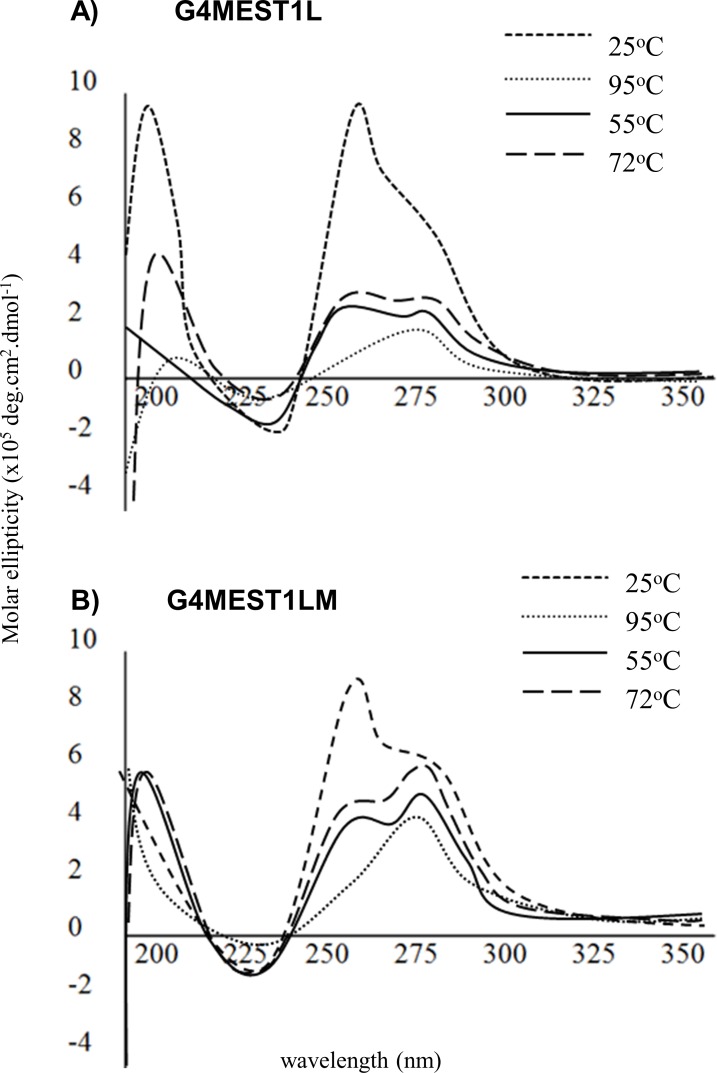
Structural comparison of G4 formation at G4MEST1L and G4MEST1LM in PCR buffer, during a mock PCR cycle. Spectral profiles were gathered at PCR relevant conditions.

For the oligonucleotides G4MEST1L and G4MEST1LM, G4 structure dissociated at 95°C, (Fig 10). No re-folding was observed for G4MEST1L at subsequent temperature stages. At 72°C G4MEST1LM (methylated) regained an elliptical peak at ~290 nm, which was equivalent in intensity to analysis at 25°C. A significant increase in ellipticity at 265 nm was also observed, however, this was less than seen at 25°C. This indicated that after denaturation the methylated oligonucleotide was able to regain G4 structural formation at 72°C.

**Fig 11:**
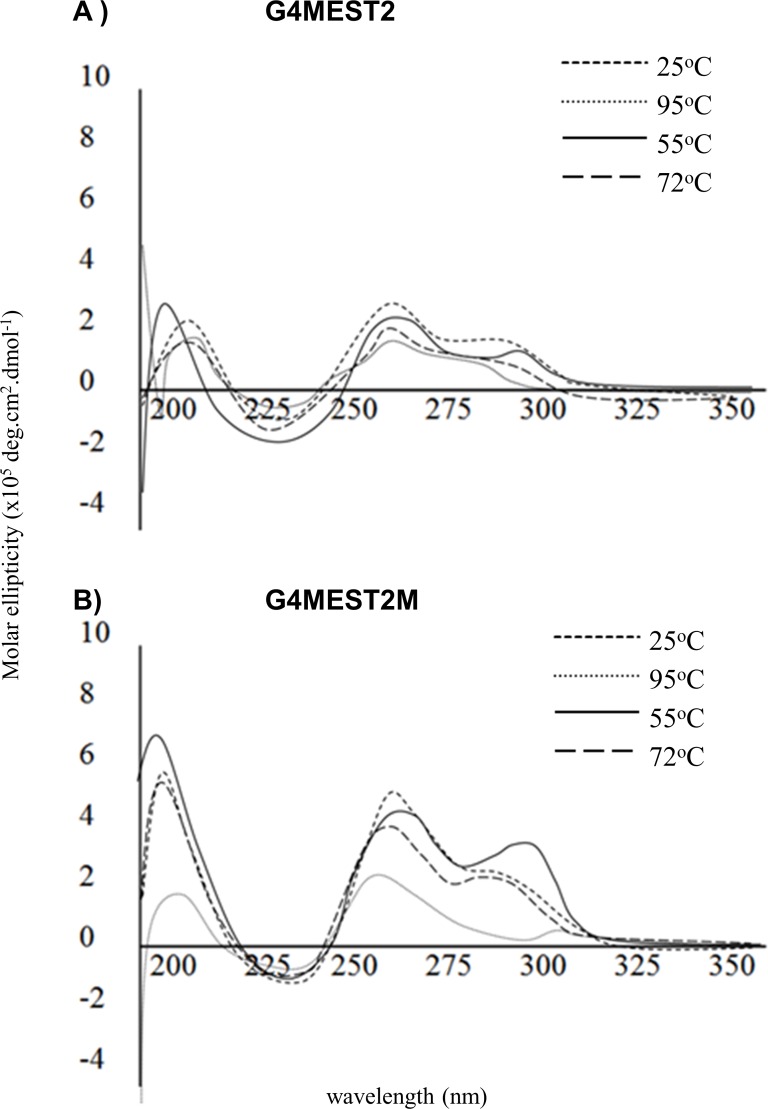
Structural comparison of G4 formation at G4MEST2 and G4MEST2M in PCR buffer, during a mock PCR cycle. Spectral profiles were gathered at PCR relevant conditions.

Due to the low elliptical maxima observed with G4MEST2 and G4MEST2M, structural change was difficult to quantify (Fig 11). Ellipticity at 290 nm was lost at 95°C and significantly reduced at 265 nm decreased for both G4MEST2M and G4MEST2. This change was more apparent for G4MEST2M, however, at 55°C G4MEST2M regained elliptical maxima at both 290 nm and 265 nm. The spectral profile at 72°C was equivalent to 55°C, indicating that the methylated oligonucleotide had regained significant structural formation during the course of the mock PCR cycle.

**Fig 12:**
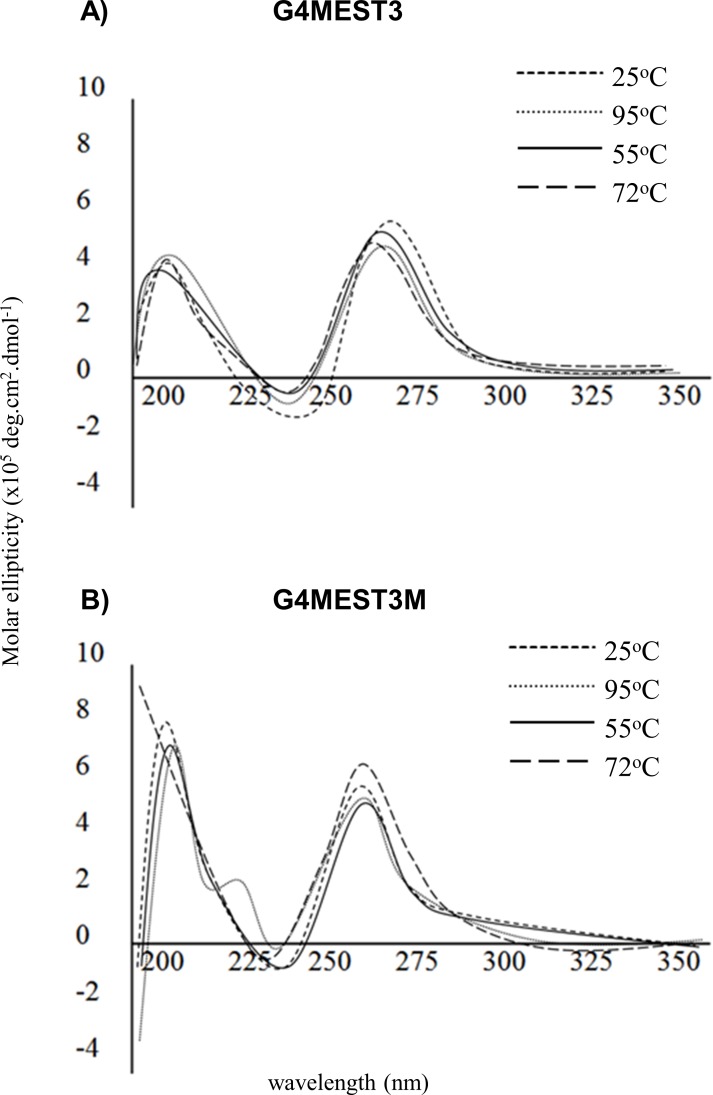
Structural comparison of G4 formation at G4MEST3 and G4MEST3M in PCR buffer, during a mock PCR cycle. Spectral profiles were gathered at PCR relevant conditions. Molar ellipticity (x10^5^ deg.cm^2^.dmol^-1^) is presented on the vertical axis and wavelength (nm) on the horizontal axis.

G4MEST3 and G4MEST3M maintained structure through the initial two minute denaturation period (Fig 12). The mock PCR was then extended for a total of seven cycles. At cycle five, G4MEST3M began to demonstrate significant loss of structure at 95°C, however, the elliptical profile was completely regained by 55°C. G4MEST3 began to dissociate at between cycle six and seven, after which, structure did not re-associate (data not shown).

## Discussion

Previously, we described a unique ADO phenomenon which occurred during PCR genotyping of the human *MEST* promoter region. Initial investigations demonstrated this was due to the combination of both cytosine methylation and formation of Hoogsteen bonds, which was indicative of G4 structure [19]. In order to confirm formation of G4 and extend our understanding of how non B-DNA structure could contribute towards ADO, we applied fluorescent footprinting techniques on a range of DNA templates, at conditions relevant to PCR.

### FADFA and CD spectroscopy performed on fluorescent oligonucleotides

The sequence of G4MESTFAM2 contains four G-tracts that range in length from 3 nt to 5 nt. Due to the differing length of G-tracts, multiple potential G4 conformations are possible in this region, however, the total number of G-tetrad layers is limited to three by G-tract 1. The spectral signature of G4MESTFAM2 (Fig 2A) suggested predominantly parallel G4 structure, but also indicated the presence of anti-parallel structures. This was reinforced by FADFA, which indicated that the two outer guanines of G-tract 2 and 3 (≥ four G repeats) are both capable of alternatively contributing to G4 structure. The apparent polymorphic nature of this region likely resulted in the decreased resolution for G4 detection.

The CD spectra of G4MESTFAM3 indicated that parallel G4 conformation was favoured (Fig 2B). G4MESTFAM3 contained seven G-tracts which could potentially contribute towards G4 formation and each G-tract consisted of either three or four guanines. FADFA demonstrated that in PCR buffer, G4 formation occurred at G-tracts 4–7, which is consistent with predictions made using QGRS mapper [23]. Cleavage occurred at both G34 and G37 from G-tract 5, which may indicate that between molecules, each terminal guanine alternatively contributes towards G4 formation. In addition to this pattern of cleavage, G4MESTFAM3 displayed alternative cleavage patterns that appeared to be induced by different ionic conditions (S1 Fig). The structural topology of G4MEST1 was not analysed using single stranded-oligonucleotides.

### Detection of G4 formation using Mung Bean Nuclease

Prior G4 analyses of the *MEST* promoter region were performed using single stranded oligonucleotides. Although suitable as a preliminary analysis, it is likely that these conditions would favour G4 over formation of other structures such as H-DNA and therefore, the results may not have been representative of dsDNA. To verify G4 formation and identify the contributing nucleotides we performed FANFA and FADFA on dsDNA templates.

It is important to use more than one analytical approach, as DMS footprinting represents an averaged cleavage pattern and highly polymorphic regions, such as G4MEST2 may be difficult to detect. Therefore, an initial screen for non B-DNA formation in dsDNA was performed using enzymatic digestion by Mung Bean nuclease. This technique utilizes single strand specific enzymatic digestion by Mung Bean endonuclease instead of chemical treatment by DMS and piperidine, as used for DMS footprinting. Mung Bean nuclease binds and cleaves single stranded DNA at the O-3’-P phosphodiester bond [24], with a preference for cleaving single-stranded loops of secondary structures [25]. Because Mung Bean nuclease does not cleave the complementary strand, both DNA strands of a dsDNA template can be used for detecting structural formations.

This method was applied to the same synthetic DNA templates that were previously used to demonstrate ADO, allowing us to directly assess structural formation in our PCR templates [19]. Cleavage closely corresponded with the previously identified motifs G4MEST1L, G4MEST2 and G4MEST3, and a novel G4 motif was identified on the C-rich strand (G4MESTA). This confirmed that the motif of G4MEST2 exhibits non B-DNA formation in dsDNA. On the C-rich strand, G4MEST3 demonstrated a single position of cleavage which corresponded to the link between G-tract 4 and 5 and this result corresponds to the position of symmetry if H-DNA or a Hoogsteen hairpin were the prominent formation [26–29].

### FADFA performed on dsDNA templates

We first investigated G4 formation after heat denaturation and rapid annealing, as occurs during the initial stage of hot-start PCR. Two predominant positions of non B-DNA formation (G4MEST1 and G4MEST3) were identified on one strand, and one (G4MESTA) was identified on the complementary strand. The pattern of cleavage at G4MEST1 and G4MESTA was indicative of G4 formation, however, G4MEST3 displayed guanine protection across G-tracts 2–7. This may reinforce earlier indications that multiple Hoogsteen based structures can form at the *MEST* promoter region. Given the slow proposed association rates of certain triplex sequences, especially at increased temperatures, G4 formation would appear more probable, however, further analysis is required to differentiate between these structures [30–32]. Guanine cleavage was not detected at G4MEST2, however, this is likely to reflect the polymorphic nature of this region. G4 formation at G4MESTA is intriguing as QGRS mapper [23] predicted a two guanine tetrad structure with a low G4 propensity (S1 Table). Our results suggested G4 formation is likely to consist of a three layered structure where a nearby guanine substitutes for the shorter G-tract 1 (Fig 7). This demonstrates a limitation in the power of bioinformatic prediction software and shows how FADFA can be a powerful technique for analysis of multiple G4 in long dsDNA templates.

We next tested dsDNA for the presence of G4 in the absence of thermal denaturation. The aim of this investigation was to determine if the denaturation stage at the start of PCR facilitated structural formation of G4 by separating the dsDNA. To do this, we investigated if non B-DNA or B-DNA formation was kinetically favoured in the absence of thermal denaturation. G4MEST3 proved to be the only motif that transitioned from B-DNA to adopt G4 formation, without strand denaturation (Fig 9). This observation was consistent for both methylated and non-methylated DNA templates and the resulting pattern of DMS cleavage was representative of G4 formation. This experiment confirms the robust nature of the G4MEST3 quadruplex. It is interesting to note the substantial difference between the DMS footprints of G4MEST3 obtained from single stranded oligonucleotides and the different treatments of dsDNA. Due to the short linkers connecting G-tract 3 with G-tract 4 and G-tract 6 with G-tract 7, this structure may adopt an antiparallel conformation in dsDNA, whereas CD spectroscopy indicated that parallel conformation was favoured in oligonucleotides.

This analysis indicated that under the correct ionic conditions G4 formation at G4MEST3 may occur in the genomic DNA template, however, formation at G4MEST1 and 2 appear to be favoured by the conditions of PCR. To investigate this further, we returned to studying G4 formation using CD spectroscopy and non-fluorescent oligonucleotides.

### G4 stability during PCR

To investigate if G4 structure was maintained at the temperatures used in a typical PCR, spectral profiles were analysed at each of the three key stages (denaturation, annealing and extension). This analysis demonstrated that at conditions relevant to PCR, the re-folding rates for methylated G4 were significantly greater than non-methylated G4 after denaturation. For all methylated oligonucleotides, structure was present at the PCR extension temperature (72°C), but no significant structure was observed for non-methylated oligonucleotides after denaturation. This observation suggests a mechanism for the interaction between G4 structures and cytosine methylation that may account for ADO during PCR. Although non-methylated G4 have higher thermal stability [19], methylation appears to enhance re-folding of G4 and this is likely to influence amplification of the maternal *MEST* allele during PCR. This observation is similar to one made by Hardin *et al*. (1993) [33]. G4 structure is likely to impair amplification by Taq polymerase [34], and this could provide a mechanism for ADO of the methylated allele during PCR genotyping.

### Conclusion

ADO at the human *MEST* promoter region was previously demonstrated to require both methylation and non B-DNA formation, where the likely structure was proposed to be G4 [19]. In the present analysis, PCR amplicons were fluorescently labelled and interrogated by FADFA and FANFA, enabling investigation of the interaction between G4 structures and cytosine methylation, in a scenario relevant to PCR and genomic DNA. We confirmed G4 formation at the motifs of G4MESTA, G4MEST1, G4MEST2 and G4MEST3 from the human *MEST* promoter region in both oligonucleotides and dsDNA templates. We demonstrated that G4 are likely to rapidly form during PCR, and the G-tracts involved in G4 structure formation were precisely mapped. However, structural formations appeared to be highly polymorphic, especially at G4MEST2 and G4MEST3, which are likely to adopt alternative non B-DNA structures, such as H-DNA. Additionally, the observed G4 topologies were highly dynamic and different topologies were observed with single and double stranded templates, and under different ionic conditions.

Although cytosine methylation did not appear to significantly alter G4 conformation, G4 re-folding after thermal denaturation occurred more rapidly in the methylated G4 compared to the non-methylated G4. This suggests that methylation has a role in aiding the kinetic association of G4 folding in vitro. If G4 on the methylated maternal allele are capable of more rapidly re-folding than those on the non-methylated allele, this could direct ADO during PCR amplification of the *MEST* promoter region, through polymerase arrest. Although it is still unclear how cytosine methylation impacts on renaturation of G4 structures, demonstrating that this phenomenon underlies the ADO observed at this locus will provide a focus for future research.

## Supporting Information

S1 Fig FADFA performed on single stranded oligonucleotide G4MESTFAM3 in NaPi buffer. Grey bars represent DMS on FAM labelled G4MESTFAM3 oligonucleotide. Nucleotide sequence is on the X-axis and relative fluorescence units are on the y-axis. A: Negative control in MPW; B: Guanine cleavage resulting from treatment of G4MESTFAM3 in NaPi + 100 mM KCl; C: Guanine cleavage resulting from treatment of G4MESTFAM3 in NaPi + 100 mM NaCl.
(DOCX)

S1 Table QGRS predictions for novel putative G4 sequences. (DOCX)

## Abbreviations

DMS: Di-methyl sulfate
G4: G-quadruplex
FADFA: Fluorescent analysis of DMS footprinting assay
FANFA: Fluorescent analysis of nuclease footprinting assay
PCR: Polymerase Chain Reaction
MEST: Mesoderm expressed sequence transcript
ADO: Allelic drop-out
CD: Circular dichroism
