# Supplementary material for: Structural Analysis of G-Quadruplex Formation at the Human MEST Promoter
Source: PLoS One. 2017 Jan 4;12(1):e0169433. doi: 10.1371/journal.pone.0169433 (PMC5214457; doi:10.1371/journal.pone.0169433)
Supplement: S1 Fig — Grey bars represent DMS on FAM labelled G4MESTFAM3 oligonucleotide. Nucleotide sequence is on the X-axis and relative fluorescence units are on the y-axis. A: Negative control in MPW; B: Guanine cleavage resulting from treatment of G4MESTFAM3 in NaPi + 100 mM KCl; C: Guanine cleavage resulting from treatment of G4MESTFAM3 in NaPi + 100 mM NaCl. (DOCX) [file pone.0169433.s001.docx]

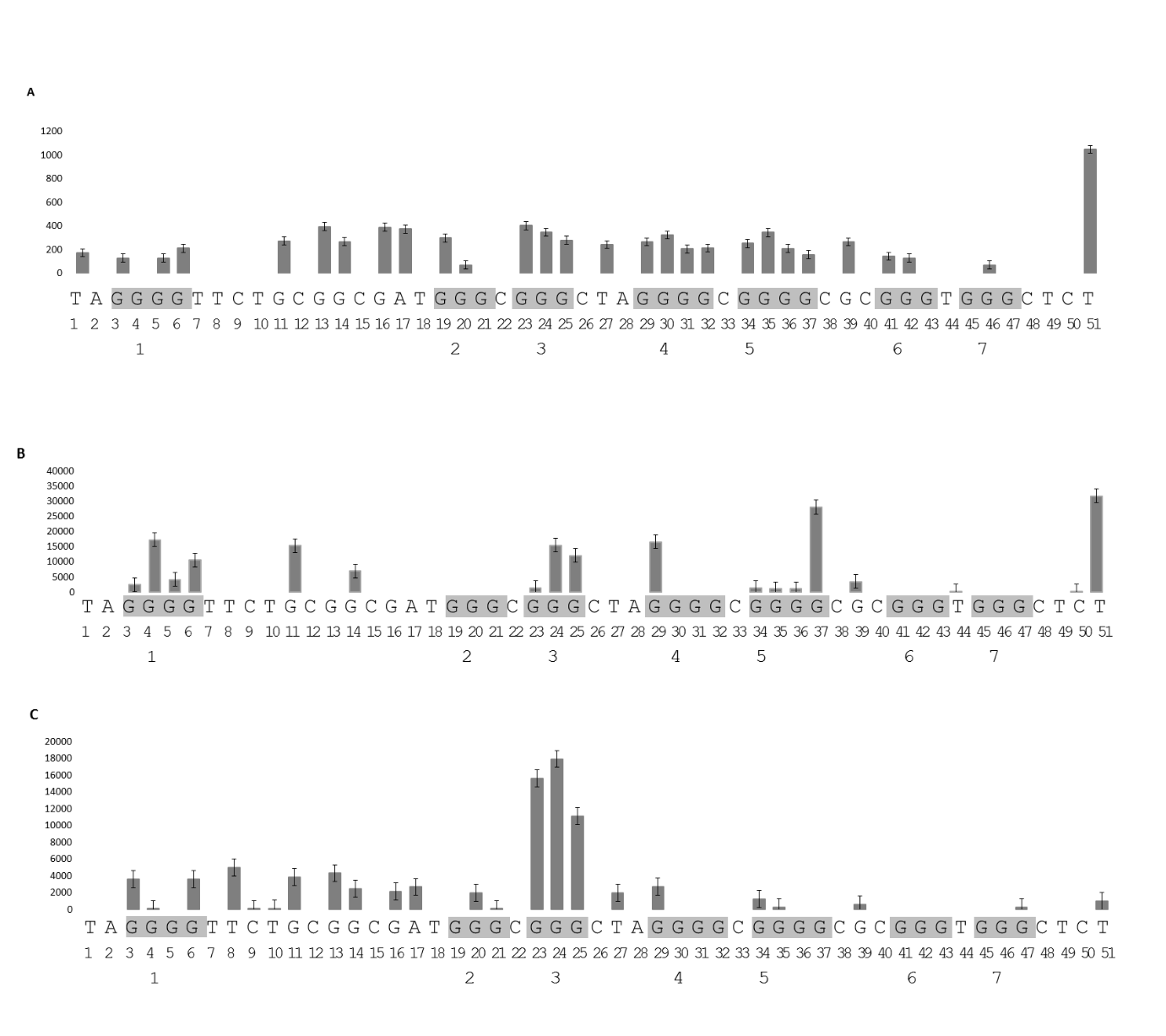


**S1 Fig. FADFA performed on single stranded oligonucleotide G4MESTFAM3 in NaPi buffer.** Grey bars represent DMS on FAM labelled G4MESTFAM3 oligonucleotide. Nucleotide sequence is on the X-axis and relative fluorescence units are on the y-axis. A: Negative control in MPW; B: Guanine cleavage resulting from treatment of G4MESTFAM3 in NaPi + 100 mM KCl; C: Guanine cleavage resulting from treatment of G4MESTFAM3 in NaPi + 100 mM NaCl.
