# Supplementary material for: Structural Analysis of G-Quadruplex Formation at the Human MEST Promoter
Source: PLoS One. 2017 Jan 4;12(1):e0169433. doi: 10.1371/journal.pone.0169433 (PMC5214457; doi:10.1371/journal.pone.0169433)
Supplement: S1 Table — (DOCX) [file pone.0169433.s002.docx]

**S1 Table. QGRS predictions for novel putative G4 sequences**

| G4 Name | Nucleotide length | Predicted G4 sequence* | G-Score |
| --- | --- | --- | --- |
| G4MESTA | 32 | GGGGCAGCAGGGCTCTGGGGTGCCGGCCGTGG | 32 |
| G4MESTA | 22 | AGGGCTCTGGGGTGCCGGCCGTGG | 34 |
| G4MESTB | 36 | GGGGAGGGTTTCTGCAGCAGAATCTCGGGCTCAGGG | 54 |

*QGRS predictions of G4 stability and formation for the motif G4MESTA. A higher G-score is indicative of higher predicted stability. Underlined guanines are predicted to contribute towards G4 formation. Bold nucleotides correspond to the position of nuclease cleavage represented in Figure 5.
